# Tinnitus, hyperacusis and somatic complaints in a diverse Norwegian clinical population: implications for assessment and rehabilitation

**DOI:** 10.1080/07853890.2026.2696613

**Published:** 2026-07-05

**Authors:** Tove Bring Solum, Andreas Falck, Kaia Ringstad Andreassen, Guri Engernes Nielsen

**Affiliations:** a Department of Special Needs Education, University of Oslo, Oslo, Norway; b Unicare Landaasen, Norway

**Keywords:** Tinnitus, hyperacusis, somatic complaints, anxiety, depression, gender

## Abstract

**Objectives:**

Patients with tinnitus often have somatic complaints, a reality that is understudied in the broader literature on tinnitus. This study aims to characterize tinnitus patients with somatic complaints, investigate associations between somatic complaints and tinnitus, hyperacusis, and symptoms of anxiety and depression, and to compare patient profiles across genders and healthcare services.

**Design:**

A cross-sectional study with a digital questionnaire consisting of Tinnitus Sample Case History Questionnaire (TSCHQ), Tinnitus Handicap Inventory (THI), Hyperacusis Questionnaire (HQ), Hospital Anxiety and Depression Scale (HADS) and Generalized Anxiety Disorder-7 (GAD-7). The somatic complaints investigated were Headaches, Dizziness, Temporomandibular Dysfunction (TMD), Neck pain and Pain Syndromes, as measured using the TSCHQ.

**Study sample:**

A total of 144 participants with tinnitus were sampled, from four hearing clinics and two rehabilitation centres in Norway.

**Results:**

Several positive associations were observed between somatic complaints and higher levels of tinnitus, hyperacusis, depression and anxiety. Moreover, somatic complaints were more common among women, and those recruited from rehabilitation centres.

**Conclusions:**

Tinnitus patients, with or without comorbid hyperacusis, have several psychological and somatic complaints that need to be mapped thoroughly. An interdisciplinary approach should be considered for patients with tinnitus and comorbid somatic symptoms.

## Introduction

Tinnitus has emerged as an increasing public health concern [1]. Langguth et al. [2] distinguish between tinnitus, the perception of sound without an external source, and tinnitus disorder, in which this perception is accompanied by significant distress and greater clinical complexity. Although many individuals experience tinnitus without major difficulties, 10–20% report substantial burden [2]. Hyperacusis, i.e. an intolerance to everyday sounds perceived as excessively loud or painful, frequently co-occurs with tinnitus and limits participation in work, leisure, and social activities [3,4]. Coexisting tinnitus and hyperacusis are linked to more somatic and psychological symptoms, including pain, dizziness, headaches, neck pain, and elevated depression scores [5,6]. These complex health profiles are necessary to understand in the context of clinical practice, in order to better provide suitable treatment pathways.

Somatic complaints among patients with tinnitus are associated with increased tinnitus severity and reduced quality of life [7–10]. Patients with headaches, for example, often report greater tinnitus severity, dizziness, and higher depression scores than those without headaches [11]. A systematic review by De Meulemeester et al. [12] found that individuals with both tinnitus and pain exhibit more psychosocial difficulties and more severe hyperacusis than those with tinnitus alone. Moderate evidence links headache to increased tinnitus severity, underscoring the importance of a multidisciplinary approach. Similarly, Deklerck et al. [13] identified significant associations between tinnitus and headaches, including migraine.

Tinnitus is associated with psychological factors such as anxiety and depression, which may both contribute to and be exacerbated by the condition, potentially reflecting shared neural mechanisms [2,14]. The literature identifies numerous factors associated with chronic tinnitus, such as psychological distress and somatic symptoms [15]. Psychological processes may contribute to the co-occurrence of tinnitus, pain, and emotional difficulties [16]. Aazh and Moore [17,18] found that one-third of tinnitus patients show abnormal or borderline abnormal depression scores and emphasized the importance of psychological screening. Other studies highlight additional psychological and somatic difficulties among tinnitus patients [19]. Anxiety and stress are also common in individuals with neck pain and TMD [10]. Coexisting TMD and tinnitus correlate with pain, anxiety, depression, and reduced quality of life [8].

Furthermore, gender differences have been observed with respect to how tinnitus and hyperacusis interact with somatic and psychological complaints. Women tend to report higher anxiety, somatization, stress, and tinnitus-related distress, while men report more bodily pain [20,21]. Seydel et al. [22] found greater somatic symptoms among older women with tinnitus. Haug et al. [23] reported strong associations between anxiety, depression, and functional somatic symptoms across genders, while Ausland et al. [24] found more somatic symptoms among women with chronic tinnitus. Younger women with tinnitus appear particularly likely to present TMD, neck pain, dizziness, and headaches, often with stress as a contributing factor [25].

Despite extensive research on the psychological aspects of tinnitus and hyperacusis [6,15,17], few studies have examined these conditions within somatic healthcare settings, where patients often present complex symptom profiles. This study therefore investigates how tinnitus and hyperacusis present and are managed within the Norwegian somatic healthcare system. Participants were recruited from rehabilitation centres and hearing clinics to provide a comprehensive characterization of tinnitus patients with somatic complaints. Specifically, the study examines associations between somatic symptoms, tinnitus, hyperacusis, anxiety, depression, and gender, and explores differences between inpatient and outpatient populations to investigate whether patients referred to rehabilitation centres have more complex health profiles compared to patients receiving treatment at hearing clinics.

## Methods

### Design

This cross-sectional study, conducted with two different samples from the Norwegian tinnitus population, aimed to examine the characteristics of the population, and explore if there were associations between tinnitus, hyperacusis, gender, symptoms of anxiety and depression and somatic complaints. A digital survey consisting of different questionnaires was used to obtain the information required to conduct the analysis. The somatic complaints investigated in this study were headaches, neck pain, temporomandibular disorder (TMD), dizziness/vertigo (from here on called dizziness), and pain syndromes.

Our main question was, what are the characteristics of patients with tinnitus (with or without hyperacusis) and somatic complaints? Specifically, we investigated the following research questions:

1. What is the relationship between hyperacusis, measured using the Hyperacusis Questionnaire (HQ), and somatic complaints?
2. What is the relationship between the degree of tinnitus distress, measured by Tinnitus Handicap Inventory (THI), and somatic complaints?
3. What is the relationship between the symptoms of anxiety and depression, measured by the Hospital Anxiety and Depression Scale (HADS-D, HADS-A) and Generalized Anxiety Disorder (GAD-7), and somatic complaints?
4. What is the relationship between gender and somatic complaints?
5. What are the differences in incidence of somatic complaints between inpatients and outpatients?

### Participants

The participants were from two different samples. The interest of the study was to look at a wide selection of the Norwegian tinnitus population, and also to compare the part of the population being inpatients at rehabilitation centres for 14 days pr stay (2 sequential stays within 4–6 months), and the part of the population coming to outpatient hearing clinics.

The first set of participants was recruited through the research project ‘Investigation of a Norwegian clinical population with tinnitus and hyperacusis’, led by Guri Engernes Nielsen at the University of Oslo. These participants were recruited between May 5, 2021, and March 31, 2022, from three public hearing clinics (Molde Hospital, St. Olavs Hospital and Ahus) and one private hearing clinic (HØR Oslo) in Norway. These clinics were chosen because they are known for having a good knowledge of tinnitus and hyperacusis. They also have a large geographical distribution in Norway. Audiologists assisted with the recruitment to the project after the participants had undergone regular audiometric tests and asked if they had tinnitus. If so, they were asked to participate in the project. Those who agreed and signed the consent form were sent a digital link to the questionnaire shortly after their visit to the clinics. 114 signed the consent form in this sample and 80 (70.2%) participants followed through with the questionnaire.

The second set of participants was recruited from two inpatient rehabilitation centres in Norway (Unicare Landaasen and Nordtun HelseRehab). They are located in two different parts of Norway. The participants were recruited from January 5th to March 31st 2023. All patients in the tinnitus program within these dates were provided information about the study. There were 89 patients who were provided with information regarding the study. Of these, 64 (71.9%) provided consent and participated.

### Measures

The following Questionnaires were administered:

*The Tinnitus Sample Case History Questionnaire (TSCHQ)* [26,27] is divided into 35 questions which, among other things, cover pain, dizziness, temporomandibular disorder, what affects tinnitus, negative feelings about tinnitus, what frequency the tinnitus has and whether you think it may have a causal explanation. The TSCHQ is used both internationally and in Norway, and the questionnaire was translated into Norwegian and validated in Norway in 2019 [27]. It has various tick boxes, both yes/no/don’t know, list of answer options, possibility to write down yourself, as well as VAS scales (0–100) For the purpose of this study, a number of variables from TSCHQ were selected for the interferential analyses: Gender (question 2), Temporomandibular Disorder (TMD) (question 33), Dizziness/vertigo (question 32, headaches (question 31), neck pain (question 34) and pain syndromes (question 35). Furthermore, a range of variables were selected to facilitate descriptive analyses.
*Tinnitus Handicap Inventory (THI****)*** [28] consists of 25 questions about tinnitus and the perceived degree of tinnitus handicap. The THI-NOR, translated and validated by Heggdal et al. [29], showed psychometric properties similar to the original THI. In this study, a revised edition of the THI, with slightly different wording for certain questions, was used. The revised version used here followed the guidelines for the translation and adaptation of questionnaires in audiology [30]. This version is found to have adequate validity and reliability (Nielsen and Falck, in preparation). The answer options are ‘Not at all’ (0 points), ‘Occasionally’ (2 points) and ‘Always’ (4 points). All points are summed up into one sum and the scale runs from 0 to 100.
*The Hyperacusis Questionnaire (HQ)* [31,32] consists of 14 questions about hyperacusis with the answer options ‘No’ (0 points), ‘Yes, occasionally’ (1 point), ‘Yes, quite often’ (2 points) and ‘Yes, often’ (3 points). All points are summed up into one sum and the scale runs from 0 to 42. The threshold value for what indicates that a patient has hyperacusis is debated and varies from 22 to 28 [31,33].
*The Hospital Anxiety and Depression Scale (HADS)* [34] was validated in Norway [32]. The questionnaire consisted of 7 questions about symptoms of anxiety and 7 questions about symptoms of depression and aimed to detect symptoms of anxiety and depression in patients with somatic illnesses. The answer options were divided into 4 under each question, and there were slightly different answer options based on the questions that give 0, 1, 2 or 3 points. The scale goes from 0 to 42 in total, with 0–21 on each of the subscales, where 0–7 is classified as within the normal range, 8–10 as mild symptoms and 11–14 as moderate symptoms and 15–21 as severe symptoms. In this study, the total score for each subscale; HADS-D and HADS-A, was used.
*The Generalized Anxiety Disorder 7 (GAD-7)* [35,36] measures Generalized Anxiety Disorder, which differs from the more general anxiety measured in the HADS-A. The scale consists of seven questions about anxiety, worries and restlessness, with four answer options for each question, graded from 0 to 3 points; not at all (0), some days (1), more than half of the days (2) and almost every day (3). Johnson et al. [35] tested the psychometric properties of the GAD-7 using a Norwegian sample. In this study, the total sum of the GAD-7 scores was used. Aazh and Moore [18] recommended GAD-7 as a good questionnaire for use in patients with tinnitus.

### Data analyses

The analyses were conducted using Jamovi 2.2.5. Analyses investigated both samples at once, with the aim of generalizing to the population of patients with tinnitus (with or without hyperacusis) in Norway. Additional complexity was introduced because the two samples were not equally representative: the sample sizes (80 and 64) don’t reflect the proportions of patients in the population being treated in rehabilitation centres, and outpatient hearing clinics, respectively. In the general population there are likely to be fewer tinnitus patients in the rehabilitation centres, than in the outpatient hearing clinics. For this reason, patient group affiliation was included as a covariate where applicable. Research questions 1–3 were investigated using bivariate correlations between somatic complaints and the HQ, THI, HADS and GAD, respectively, with patient group partialled out. Research question 4 was investigated using bivariate Χ^2^-analyses, and research question 5 was investigated using bivariate Χ^2^-analyses together with a Mann-Whitney U-test.

As the five somatic complaints were investigated in separate analyses for each research question (with the exception of one test), we acknowledge a risk for false positive findings due to multiple comparisons. Therefore we consider statistical tests at two different alpha values for the analyses involving individual somatic complaints: both the standard .05, and the more conservative limit .01 (corresponding to Bonferroni-corrected alpha = .05). P-values above .01 are interpreted with more caution (Table 1).

**Table 1.** Descriptive data, both samples.

	Total	Outpatient group	Inpatient group
Number of participants	144	80	64
Men	71	43	28
Women	73	37	36
Age	53 (SD = 11.90)	51.5 (SD = 13.8)	54.8 (SD = 8.76)
Years with tinnitus	10.8 (SD = 10.60)	10.6 (SD = 11.2)	11.1 (SD = 9.70)
HADS-D	5.40 (SD = 4.65)	3.65 (SD = 3.70)	7.59 (SD = 4.81)
HADS-A	7.22 (SD = 4.87)	5.44 (SD = 4.01)	9.44 (SD = 4.96)
GAD-7 score	6.42 (SD = 4.97)	4.86 (SD = 4.29)	8.38 (SD = 5.09)
THI score	44,8 (SD = 25.20)	32.4 (SD = 21.40)	60.3 (SD = 20.90)
HQ score	18.7 (SD = 10.40)	13.8 (SD = 8.85)	24.8 (SD = 8.95)
Conscious of tinnitus % of the time	61.3 (SD = 28.90)	51.5 (SD = 28.90)	73.5 (SD = 23.9)
Annoyed with tinnitus % of the time	35.00 (SD = 29.40)	28.8 (SD = 27.10)	42.7 (SD = 30.60)
Hearing problem	65.30 % (*N* = 94)	60.00 % (*N* = 48)	71.88 % (*N* = 46)
Use of hearing aid	31.25 % (*N* = 45)	11.25 % (*N* = 9)	56.25 % (*N* = 36)
Varying tinnitus sound	68.10 % (*N* = 98)	58.75 % (*N* = 47)	79.69 % (*N* = 51)
Tinnitus all the time	85.40 % (*N* = 123)	77.50 % (*N* = 62)	95.31 % (*N* = 61)
Psych. treatment	14.58 % (*N* = 21)	10.00 % (*N* = 8)	20.31 % (*N* = 13)
Tried one or more treatments for tinnitus	54.17 % (*N* = 78)	31.25 % (*N* = 25)	82.81 % (*N* = 53)
Headaches	44.4% (*N* = 64)	27.5% (*N* = 22)	65.6% (*N* = 42)
Dizziness	40.3% (*N* = 58)	25.0% (*N* = 20)	59.4% (*N* = 38)
TMD	29.2% (*N* = 42)	17.5% (*N* = 14)	43.8% (*N* = 28)
Neck pain	57.6% (*N* = 83)	38.8% (*N* = 31)	81.3% (*N* = 52)
Pain syndromes	45.8% (*N* = 66)	30.0% (*N* = 24)	65.6% (*N* = 42)

### Ethical approval and ethical concerns

This study was approved by the Norwegian Regional Ethics Committee (REK, Reference Number 178677). We followed the guidelines for research with voluntary participation. The respondents had to sign an informed consent form, and the data were safely stored and processed on a secure server at the University of Oslo.

The research was performed in accordance with the principles stated in the Declaration of Helsinki.

## Results

### Participants characteristics

### Associations between somatic complaints and hyperacusis, tinnitus distress, depression, and anxiety (Research questions 1–3)

Partial Pearson’s correlations were computed between each of the somatic complaints (headache, dizziness, TMD, neck pain and pain syndromes) and HQ, THI, HADS-D, HADS-A and GAD, respectively, with patient group partialled out. For each somatic complaint, we also reported means and standard deviations for each measure separately for participants who reported the complaint, and those who did not report the complaint, respectively.^1^ These statistics are presented in Table 2. Correlations between somatic complaints and the different measures had exclusively positive signs, and most were significant at the α = .05 level. If we constrain our interpretation to the more conservative α = .01 level, then three relations pass the threshold: Headache and HQ scores, Dizziness and HADS-D scores, and Pain syndromes and GAD scores (Table 2). HADS-A shows weaker associations with somatic complaints overall, and most of the relations involving HADS-A scores were not significant.

**Table 2.** Correlations, including 95% confidence intervals, between the somatic complaints and measures of tinnitus, hyperacusis and psychological symptoms, with patient group partialled out. Included are also means and standard deviations of the respective scores among patients with, and without, each complaint.

Somatic complaints		HQ		THI		HADS-D		HADS-A		GAD	
Symptom	Present?	M(SD)	*r*_p_ [95% CI]	M(SD)	*r*_p_ [95% CI]	M(SD)	*r*_p_ [95% CI]	M(SD)	*r*_p_ [95% CI]	M(SD)	*r*_p_ [95% CI]
Headaches	Yes	23.8 (9.6)	.304***	53.7 (23.6)	.138	6.8 (5.4)	.140†	8.7 (5.2)	.133	8.1 (5.6)	.203*
	No	14.6 (9.2)	[.15, .45]	37.7 (24.4)	[−.03, .30]	4.3 (3.5)	[−.02, .30]	6.1 (4.3)	[−.03, .29]	5.1 (4.0)	[.04, .36]
Dizziness	Yes	23.1 (10.3)	.197*	55.4 (23.9)	.197*	7.3 (5.2)	.221**	8.6 (5.1)	.098	8.3 (5.4)	.210*
	No	15.7 (9.4)	[.03, .35]	37.6 (23.7)	[.03, .35]	4.1 (3.8)	[.06, .37]	6.3 (4.5)	[−.07, .26]	5.2 (4.2)	[.05, .36]
TMD	Yes	22.5 (11.2)	.103	55.0 (24.6)	.127	7.4 (5.2)	.183*	8.9 (5.8)	.125	8.6 (6.2)	.198*
	No	17.2 (9.7)	[−.06, .26]	40.6 (24.4)	[−.04, .29]	4.6 (4.1)	[.02, .34]	6.5 (4.3)	[−.04, .28]	5.5 (4.1)	[.03, .35]
Neck pain	Yes	21.9 (10.1)	.180*	52.8 (23.6)	.180*	6.7 (5.0)	.174*	8.6 (4.8)	.188*	7.8 (5.2)	.197*
	No	14.4 (9.4)	[.02, .33]	33.9 (23.4)	[.02, .33]	3.7 (3.6)	[.01, .33]	5.3 (4.4)	[.02, .34]	4.6 (3.9)	[.03, .35]
Pain syndromes	Yes	22.1 (10.0)	.137	54.6 (23.0)	.207*	6.6 (5.0)	.101	8.6 (5.1)	.145†	8.5 (5.5)	.292***
	No	15.9 (9.9)	[−.03, .29]	36.5 (24.2)	[.04, .36]	4.4 (4.2)	[−.06, .26]	6.0 (4.4)	[−.02, .30]	4.7 (3.7)	[.13, .44]

**Note:** rₚ = partial Pearson’s *r*, after controlling for the influence of patient group. †p < .10, *p < .05, **p < .01, ***p < .001.

### Do women and men differ in the prevalence of somatic complaints? (Research question 4)

The proportions of somatic complaints in the respective groups are listed in Table 3, along with tests of proportion differences. As the proportions of men and women did not differ between the two patient groups (Χ^2^_(1,_ *_N_*_=144)_ = 1.42, *p* = .233), we did not control for patient group in the statistical tests of these associations. All complaints were significantly more prevalent in women at the α = .05 level, except dizziness (*p* = .587). When applying the more conservative α = .01 level, only TMD had a significant association with gender.

**Table 3.** Differences in the prevalence of somatic complaints between the genders.

Somatic complaint	Incidence of complaint (% yes-answers within each gender)		Χ^2^ (1, *N* = 144)	*φ*
	Women (*N* = 73)	Men (*N* = 71)		
Headaches	53.4 % (*N* = 39)	35.2 % (*N* = 25)	4.84	.183*
Dizziness	42.5 % (*N* = 31)	38.0 % (*N* = 27)	.295	.045
TMD	39.7 % (*N* = 29)	18.3 % (*N* = 13)	7.99	.236**
Neck pain	67.1 % (*N* = 49)	47.9 % (*N* = 34)	5.45	.195*
Pain syndromes	54.8 % (*N* = 40)	36.6 % (*N* = 26)	4.79	.182*

Note: *p < .05, **p < .01.

### Do inpatient tinnitus patients at rehabilitation centres have a higher incidence of somatic complaints than the ones seeking outpatient hearing clinics? (Research question 5)

The proportion of somatic complaints in each group is listed in Table 1. All complaints show a strong association with patient group, with the inpatient group consistently having higher prevalence of the somatic complaints (Χ^2^s _(1,_ *_N_*_=144)_ ranging between 11.9 and 26.3, all ps < .001). Phi (φ) coefficients for the corresponding associations were as follows: φ_Headache_ = .381, φ_Dizziness_ = .348, φ_TMD_ = .287, φ_Neck pain_ = .427, and φ_Pain syndromes_ = .355.

To further investigate how the prevalence of somatic complaints differed between the patient groups, we also investigated the total number of somatic complaints (headache, dizziness, neck pain, pain syndromes and temporomandibular disorders) in the two groups. The number of complaints ranged from 0 to 5 in both groups; however, the median number of complaints was three in the inpatient group vs. one in the outpatient group, a significant difference (Mann-Whitney *U* = 954, *p* <.001, *r*_b_= .63).

## Discussion

In this study, the aim was to look at some somatic complaints in two samples of the Norwegian tinnitus patient population, and how the somatic complaints, gender, THI scores, HQ scores and symptoms of anxiety and depression (GAD-7 scores, HADS-A and HADS-D) were associated. A secondary aim was to investigate whether the two samples differed in terms of somatic complaints. First, the descriptive results are briefly discussed, then the five research questions are discussed in turn.

### Prevalence of somatic complaints in our sample

Several somatic complaints are observed to be commonly experienced among our respondents. Dizziness was prevalent, particularly so in the inpatient group, with almost three times the number of patients reporting dizziness compared to the outpatient group. A substantial percentage of respondents also had neck pain and pain syndromes. There was no information on specific diagnoses in this study, only symptoms, which means that behind the symptoms, there could be different diagnoses that have been shown to often coexist with tinnitus, such as fibromyalgia and neck injuries [37,38].

### RQ1: What is the relationship between hyperacusis, measured by HQ, and somatic complaints?

The HQ-scores were related to headaches, dizziness and neck pain at the α = .05 level. However, when applying Bonferroni-correction only the relationship with headaches remained significant (corrected α = .05, uncorrected α = .01). The findings are in line with that of other studies. De Meulemeester et al. [12] found that hyperacusis is more severe in patients who experience co-existing symptoms such as TMD (which we did not observe), headaches, neck pain as well as tinnitus. Schecklmann et al. [6] found headache, jaw disorder, general pain syndromes, vertigo and neck pain to be associated with hyperacusis, albeit only measured by one question in the TSCHQ; ‘Do sounds cause you pain or physical discomfort?’

These results also align well with the authors’ observations from clinical practice. Many patients with hyperacusis report various somatic complaints. However, the causal mechanisms underlying these associations remain unclear. One possible hypothesis is that hyperacusis may lead to increased muscular tension, which in turn can result in symptoms such as headaches, neck pain and dizziness. It has also been observed that stress can exacerbate the severity of hyperacusis. Consequently, further research is needed to clarify the relationship between hyperacusis, somatic symptoms and stress.

### RQ2: What is the relationship between the degree of tinnitus distress, measured by THI, and somatic complaints?

The THI scores correlated positively and with dizziness, neck pain, and pain syndromes on the α = .05 level. However, none of the relationships survived a Bonferroni correction. Cautiously interpreting the relationships that were significant if we do not correct for multiple comparisons, we observe that they are in line with other research [11–13]. While headaches were not significantly related to tinnitus in our sample (regardless of choice of alpha level), we might still not have been surprised to find that association based on previous research. For example, a review paper by De Meulemeester et al. [12] reported positive associations between tinnitus severity and headaches in two out of three reviewed studies, and the review by Deklerck et al. [13] found the corresponding association in eight out of nine studies. However, all the studies reviewed by De Meulemeester and colleagues and Declerck and colleagues investigated larger samples (>800 participants), enabling these studies to detect effects of much smaller size than is the case for the present study. Related to this point, the non-significant difference in THI scores for patients with and without headache was 16 points in our study, which can be compared to the significant differences in the same direction ranging between 5 and 18 in the two studies cited by de Meulemeester and colleagues [11,39].

### RQ3: What is the relationship between symptoms of anxiety and depression, measured by HADS-D, HADS-A and GAD-7, and somatic complaints?

Depression symptoms measured using the HADS-D were positively correlated with dizziness, TMD, and neck pain at the α = .05 level, but only the relationship with dizziness survived the Bonferroni correction. For anxiety, the associations differed depending on the scale used: GAD showed numerically stronger associations than HADS-A, the latter for which most were not significant. All relations between somatic complaints and anxiety symptoms were in the expected direction (positive). To the authors’ knowledge, this study seems to be the first to investigate the difference between the correlations of GAD-7 and HADS-A with somatic symptoms. The results suggest that GAD is a more sensitive measure when applied to tinnitus and/or hyperacusis populations.

Moreover, GAD-7 measures symptoms of generalized anxiety disorder, while HADS-A is designed to measure anxiety symptoms in patients with somatic illnesses. It seems that more people in this study had symptoms of generalized anxiety disorder together with somatic complaints, while those who had general anxiety symptoms measured by the HADS-A did not have these somatic complaints. Perhaps it could also indicate poorer sensitivity of the HADS-A to pick up symptoms of anxiety in this population, and perhaps it picks up more general stress reactions. Aazh and Moore [18] indicated that the GAD-7 is one of the recommended questionnaires for patients with tinnitus. However, they did not compare HADS-A with GAD-7 in the same study. The GAD-7 was found to be liked by the patients, as well as having a good sensitivity to detect symptoms of anxiety in this population [18]. Notably, the vast majority of guidelines for the diagnosis and treatment of tinnitus recommend HADS-A as the tool for investigating comorbid anxiety [40]. Our findings, together with those of Aazh and Moore [18], suggest that clinicians working with tinnitus patients should direct more attention to the choice of measure of anxiety.

The HADS-D was significantly associated with dizziness (Bonferroni-corrected), and also TMD and neck pain if we consider uncorrected p-values. This coincides with clinical experience, where patients have said that being dizzy can affect mental health by making them anxious about becoming dizzy, and that one can become depressed by withdrawing from many activities and social life, as the findings from both the GAD-7 and HADS-D suggests. The association with TMD and neck pain might be due to distress, clenching of the jaws/teeth and having more muscular tension when experiencing more symptoms of depression [41].

The results of this study imply that those who work with tinnitus patients should also have a certain focus on somatic symptoms, how to deal with them, and at the same time how to work with anxiety and depression. It seems that an interdisciplinary team is needed, as other researchers have argued [12]. It may also mean that if you have a patient with tinnitus, you should also map symptoms of anxiety and depression if you have not already done so in the first place, something previous research also encourages to do, regardless of somatic complaints [17]. The descriptive data also show that 14,58% of the total sample was in contact with psychiatric care, and that there were respondents with various symptoms of anxiety and depression in the samples.

### RQ4: What is the relationship between gender and somatic complaints?

The results of this study demonstrate a clear gender difference in the prevalence of somatic complaints, with women reporting significantly more symptoms than men across all categories except dizziness (uncorrected p-values). When applying Bonferroni-correction, only the TMD remained significantly related to gender. These findings align with previous population-based research in Norway [23,24] and suggest that gender plays a consistent role in the experience and reporting of somatic symptoms. The results also correspond with the findings of Boecking et al. [20], who reported that female tinnitus patients exhibited higher levels of somatization symptoms, including headaches.

The most pronounced gender difference was observed for temporomandibular disorder (TMD), where the prevalence among women was more than twice that of men (39.7% vs. 18.3%), consistent with earlier findings [25,42]. Other complaints such as neck pain, headaches, and generalized pain syndromes also showed moderate effect sizes (φs ranging from .182 to .236), indicating meaningful gender-related differences.

A Norwegian population study reported more somatic symptoms in women with chronic tinnitus than in men with chronic tinnitus [24]. Interestingly, Niemann et al. [21] found that male patients reported higher levels of bodily pain associated with chronic tinnitus than did female patients. However, the males in the Niemann study rated their overall health as better than the females. Female patients in their study had higher levels of stress and tension. The latter might influence clenching of the teeth and jaws, further increasing the risk for TMD in females.

These disparities may reflect both biological and psychosocial factors. Women’s higher likelihood of consulting healthcare services for symptom-related concerns may also contribute to increased reporting, although population-based studies may help mitigate potential selection bias.

### RQ5: What are the differences in incidence of somatic complaints between inpatients and outpatients?

All five somatic complaints (headache, TMD, dizziness, neck pain and pain syndromes) differed significantly between the two patient groups, even after applying Bonferroni correction. Each of the five somatic complaints was more than twice as likely to occur for a member of the inpatient group, as compared to the outpatient group. The patients in the rehabilitation centres had the most somatic complaints, both in terms of the prevalence and total number of complaints.

This may be because patients who are at rehabilitation centres for their tinnitus have more complex complaints and may have tried more types of treatment before coming to a rehabilitation centre, than those who are in the outpatient group, as the descriptive data shows. Due to Norway’s different treatment options, there are a few inpatient rehabilitation centres that admit patients with tinnitus, as well as outpatient hearing clinics. Doctors might refer the patients to an outpatient clinic first, before referring to a rehabilitation centre, or assess the complexity of the patient and refer to a rehabilitation centre as the first option when needed.

Although the self‑reported questionnaire data indicate a potential need for multidisciplinary care among patients with tinnitus, no objective information is available regarding whether participants actually sought or required consultation with relevant clinical specialists.

At such rehabilitation centres, they have a variety of classes and some individual consults regarding psychoeducation and therapeutic work on stress, cognitive behavioural therapy and Acceptance and Commitment Therapy (ACT) as well as psychoeducation on improving sleep, relaxation techniques, and jaw-, neck- and shoulder exercises. Meeting others with the same challenges is important to the participants, and they also have physical activities, focus exercises and information about hearing aids and technical aids. The authors of the present study are not aware of any other study that directly compares these two populations.

### Recommendations for further research

More research on tinnitus and somatic complaints is needed, perhaps using a questionnaire consisting of more somatic complaints than the TSCHQ. It would also be interesting to conduct a study to determine whether there are any differences in somatic complaints after tinnitus and hyperacusis treatment.

### Limitations

As the present study is cross-sectional, causal relationships cannot be inferred. The total sample size was relatively small (*N* = 144), leading many of the comparisons we make to be underpowered (especially factoring in Bonferroni corrections). However, the observed effect sizes are not unreasonable in comparison to previous research with substantially larger sample sizes (e.g. [12,13]), and it may be noted that associations generally went in the expected direction even when not statistically significant. This may limit the representativeness of the findings and reduce the generalizability of the results to broader populations.

The TSCHQ contains only binary questions about the presence or absence of each somatic complaint, consequently it does not provide information regarding the duration or severity of complaints. Moreover, no physician-verified diagnostic data or assessments of functional impairment were available, as all information was obtained exclusively through self-reported single-item measures. This may limit the scope of our conclusions.

Two of the questions in the TSCHQ pertained to TMD and pain syndromes. After these data were collected, it was discovered that these two questions had a different wording than the newer Norwegian validated TSCHQ. This may have affected the results. However, because both samples had the same questions, the results comparing the samples would be comparable. Some researchers believe that pain should be mapped using questionnaires other than the TSCHQ [12], which might indicate that the TSCHQ does not adequately cover enough pain complaints. Some in the rehabilitation centre sample also said that questionnaires like this gave them more headaches, fatigue and dizziness. These issues may have influenced who volunteered to participate in the study.

The study was based on two different samples from two subpopulations of patients with tinnitus; an inpatient group and an outpatient group. This provides more width compared to if only one sample has been used. However, it affects how the results can be generalized to a wider population of patients with tinnitus. As the samples were not stratified, the proportion of inpatients was likely to be greater in the total sample than in the population. In order to still be able to generalize the results to a wider population, group affiliation was included as a covariate in the statistical analyses. This causes the correlations between variables to be calculated as if the value of the group affiliation variable was the same for all participants, which means that the difference between the groups has less of an impact on the statistical calculations. This adjustment does not eliminate all problems with generalization, and the sample is therefore not completely representative. The advantage of the two samples is that one obtains a more comprehensive picture of the associations that exist among tinnitus patients with different degrees of discomfort. The samples are also geographically spread throughout Norway, which aids in generalizability.

## Conclusions

This study addresses an interesting gap in the literature on tinnitus by mapping out the extent to which tinnitus patients suffer from somatic complaints, and how these complaints interact with other symptoms, while also comparing patient profiles across different levels of somatic healthcare services. The study demonstrated several associations between tinnitus, hyperacusis, gender, symptoms of anxiety and depression, and somatic complaints. The results highlight a need for screening for somatic comorbidities, which are often associated with symptom severity and psychological distress. To more effectively capture psychological distress, such as anxiety, within this comorbid context, it is worth considering whether future guideline revisions should address the potential role of GAD-7 as a standard screening tool. Further comparative research on GAD-7 and HADS-A as screening tools for tinnitus patients could be informative.

The study highlights substantial differences between the challenges faced by tinnitus patients in inpatient rehabilitation settings and those typically encountered in standard hearing clinics, suggesting a broader need for access to a more intensive interdisciplinary treatment.

Building on this, the findings suggest a potential value in developing multidisciplinary care models that incorporate recommendations within the audiologist’s and other hearing professionals’ scope of practice, together with somatic and psychological perspectives in both screening and treatment planning. Such an approach may help ensure that comorbid conditions are more effectively identified and managed.

## Data Availability

The data that support the findings of this study are available upon request from the corresponding author, T.B.S.

## Note

1 We did not include t-tests of the mean differences, as the included tests of the partial correlation coefficients convey the same information.
